# Warfarin related nephropathy: a case report and review of the literature

**DOI:** 10.1186/s12882-016-0228-4

**Published:** 2016-02-01

**Authors:** Chee Yong Ng, Chieh Suai Tan, Chee Tang Chin, See Lim Lim, Ling Zhu, Keng Thye Woo, Puay Hoon Tan

**Affiliations:** Department of Renal Medicine, Singapore General Hospital, 20 College Road, Academia, Level 3, Singapore, 169856 Singapore; Department of Pathology, Singapore General Hospital, Singapore, Singapore; National Heart Centre Singapore, Singapore, Singapore

**Keywords:** Warfarin related nephropathy, Mechanical valve, Anticoagulation

## Abstract

**Background:**

Warfarin related nephropathy is one of the potential complications of warfarin therapy. Despite the well described histological entity, the clinical course and approach to warfarin related nephropathy in patients requiring life-long anticoagulation is however not well described in the literature.

**Case presentation:**

We report the clinical course of a 56 years old Chinese lady who presented with over anti-coagulation and acute kidney injury while on warfarin therapy for permanent atrial fibrillation and mechanical valve replacement. Renal biopsy was performed as the acute kidney injury was persistent despite normalization of the International Normalized Ratio and the diagnosis of warfarin related nephropathy was made. Temporary interruption of anti-coagulation, in combination with oral N-acetylcysteine resulted in subsequent stabilization of renal function.

**Conclusion:**

The diagnosis of warfarin induced nephropathy should be considered in patients presenting with unexplained acute kidney injury and over anti-coagulation. Awareness of this clinical entity is important for clinician managing anti-coagulation therapy and renal function should be monitored regularly in patients who are on warfarin therapy.

## Background

The potential deleterious effect of warfarin in patients with chronic kidney disease (CKD) was first observed in the 1970s. Warfarin, when given in anticoagulant dose as part of the “Melbourne cocktail” for the treatment of IgA nephropathy, was noted to result in an increase in urinary red blood cell counts [1]. Subsequently in 2009, Brodsky et al. reported a case series of 9 CKD patients with biopsy proven acute kidney injury during warfarin therapy [2]. The documented histological finding of acute tubular injury in the presence of glomerular hemorrhage became the hallmark feature of warfarin related nephropathy (WRN). An animal model validating WRN was subsequently reported [3] and retrospective analysis of databases of patients receiving warfarin therapy revealed that WRN could occur in patients without CKD and was associated with accelerated progression of CKD when above therapeutic range [4–6].

Despite the well described clinical entity, the clinical course of WRN remains unclear. In the original case series reported by Brodsky, four patients remained dialysis dependent and many did not recover despite normalization of the international normalized ratio (INR). Moreover, the outcome of WRN in patients where anti-coagulation should not be stopped permanently, such as in patients with mechanical heart valve, is unclear. We report the clinical course of a patient with WRN where anti-coagulation could not be stopped permanently.

## Case presentation

A 56 years old diabetic chinese lady with atrial fibrillation and mechanical mitral valve replacement (Bileaflet tilting disc valve, St Jude) was found to be over anti-coagulated [international normalized ratio (INR) of 4.95] during a routine follow up. She had been on warfarin therapy since 2001 for permanent atrial fibrillation and remained on anticoagulation after her valve replacement surgery for severe mitral stenosis secondary to rheumatic heart disease in 2008. Her medications included warfarin 3 mg OD, digoxin 250 mcg OD, bisoprolol 1.25 mg OD, fenofibrate 300 mg OD, furosemide 20 mg OD, potassium chloride 600 mg OD, glipizide 2.5 mg BiD and metformin 850 mg BiD. There were no recent changes in her medication and diet. On physical examination, she was well with no overt signs of bleeding. Her blood pressure was 130/70 mm Hg with a heart rate of 60 beats per minute. Fundoscopy did not reveal the presence of any hemorrhages or diabetic retinopathy.

The serum creatinine was noted to be elevated at 317 μmol/L, a five-fold increase from her last measured serum creatinine of 72 μmol/L 6 months ago. The provisional diagnosis was acute kidney injury (AKI) in the background of over anti-coagulation and diabetes mellitus. Further investigations were performed to elucidate the cause of her acute kidney injury. Urine microscopy revealed microscopic hematuria (Red blood cell 675/UL). Ultrasonography and computed tomography showed normal size kidneys with no evidence of urinary calculi or obstruction respectively. Autoimmune markers including anti-neutrophil cytoplasmic antibody, antinuclear antibody, anti-double stranded DNA and anti-glomerular basement membrane antibody were negative and there was no hypocomplementemia. Her urine culture was negative and 24-hour urine protein was 0.35 g/day.

**Fig. 1:**
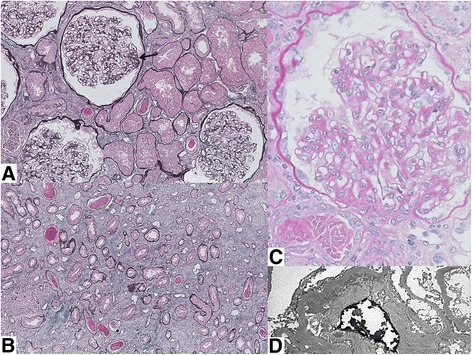
**a**. Masson trichrome-silver stain shows a glomerulus with a small segment of sclerosis (*arrow*) that is adherent to the Bowman’s capsule. A few tubules around the glomerulus show luminal red cell casts. **b**. Low magnification of several tubules containing luminal red cell casts. **c**. High magnification of a glomerulus demonstrating segmental mesangial matrix expansion and increased mesangial cells. **d**. Electron micrograph shows paramesangial electron dense deposits

**Fig. 2:**
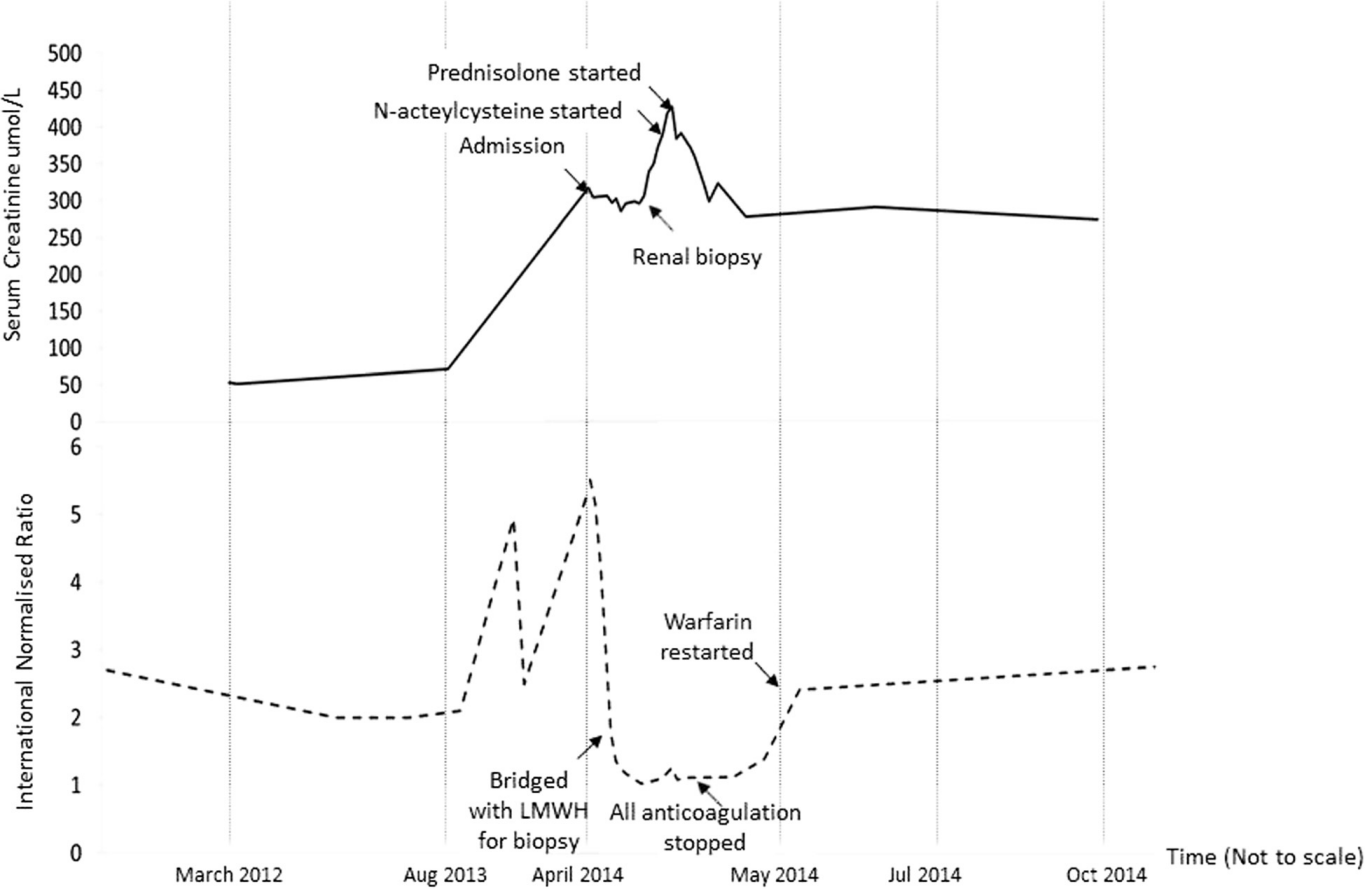
INR and serum creatinine trend of the patient

Warfarin was initially suspended to restore a therapeutic INR level. Decision was subsequently made for a renal biopsy 12 days after admission due to persistent AKI. Histology showed focal segmental and global glomerulosclerosis with mesangial hypercellularity but no crescents. The interstitium was edematous with presence of acute tubular necrosis. Specifically, a number of tubules demonstrated red cell casts, which were consistent with warfarin related nephropathy (Fig. 1). Immunofluorescence was not performed due to inadequate tissue sample. Electron microscopy, carried out on appropriately reprocessed material derived from the paraffin block, showed several electron dense deposits in the mesangial and paramesangial zones, suggestive of underlying IgA nephropathy. All anticoagulation therapy was interrupted for 5 days post renal biopsy due to the development of perinephric hematoma. Oral acetylcysteine 1.2 g BiD, followed by oral prednisolone 30 mg OD was started when the serum creatinine increased to 428 μmol/L. The serum creatinine improved to 274 μmol/L with the above interventions. The patient was bridged with low molecular weight heparin before being restarted back on warfarin, with a target INR of 2–2.5. The serum creatinine and INR 3 months post discharge were 274 μmol/L and 2.1 respectively. The INR and creatinine trends are as summarized in Fig. 2.

## Discussion

As illustrated in our patient, WRN is an important differential diagnosis in the evaluation of unexplained AKI in patients on warfarin therapy, especially in the presence of over anti-coagulation and microscopic hematuria. Although the renal biopsy showed background Ig A nephropathy, the patient had normal renal function prior to admission. The AKI was probably precipitated by over anti-coagulation, which was consistent with what was reported in the literature.

The management of WRN in patients where anti-coagulation should not be stopped permanently poses a management dilemma. The lifetime risk of mechanical valve thrombosis following permanent cessation of warfarin is exceedingly high and potentially life-threating [7]. As of now, the only novel oral anticoagulant (NOAC) drug that has been studied in patients with mechanical valves is dabigatran. In the RE-ALIGN study, dabigatran, as compared with warfarin, was associated with excess thromboembolic and bleeding events and as such is currently not indicated for use in patients with mechanical valve replacements [8]. Moreover, WRN has also been reported with dabigatran in animal models [9]. Intuitively, temporary interruption of anti-coagulation may ameliorate glomerular bleeding and result in stabilization of the renal function; however, the risks of thromboembolic events need to be taken into consideration. Extrapolating experience from management of intracranial hemorrhage in patients with mechanical heart valve, the incidence of thromboembolic events without anticoagulation in patients with mechanical heart valve was estimated to be 0.06 % per day [10]. Indeed, the European Stroke Initiative recommended that patients with very high risk for thromboembolic events should be restarted on warfarin after 10 to 14 days after intra-cranial hemorrhage [11]. In our patient, anticoagulation was interrupted for 5 days in view of post biopsy hematoma and this might have limited the extent of glomerular bleeding and renal dysfunction.

While glomerular hematuria is the inciting event in WRN, the dominant mechanism of AKI was secondary to tubular obstruction by red blood cell casts and the increased oxidative stress in the kidney. N-acetylcysteine is a well-characterized antioxidant, which has been used for many years in experimental research. Specifically, it has been shown to prevent increases in creatinine in a dose-dependent manner in animal models of warfarin induced AKI [12] and was used without any adverse reaction in our patient. We postulated that temporary interruption of anti-coagulation in conjunction with N-acetylcystine might have resulted in the stabilization of renal function that was observed in our patient.

The anti-inflammatory effect of steroids may also be useful in mitigating the onset of interstitial fibrosis as a consequence of WRN. Specifically in our patient, the beneficial effect may be secondary to its impact on the concurrent IgA nephropathy and interstitial edema. While early steroid administration was reported to accelerate recovery of AKI after gross haematuria in IgA nephropathy [13], the use of prednisolone in WRN is not strongly supported in experimental models and is limited to a single prior case report [14]. Nevertheless, based on our anecdotal evidence, the use of steroids in WRN requires further validation.

## Conclusion

In conclusion, renal function should be monitored in patients on anticoagulation therapy and the diagnosis of WRN, especially in the presence of over anticoagulation, should be considered in patient with unexplained AKI. Temporary interruption of anticoagulation in combination with N-acetylcystine may result in stabilization of the renal function. Low dose corticosteroids may be added as it can potentially suppress the inflammatory response following glomerular hemorrhage and tubular obstruction in the kidney.

## Consent

Written informed consent was obtained from the patient for publication of this case report and accompanying images. A copy of the written consent is available for review by the Editor of this journal.

## Abbreviations

CKD: chronic kidney disease
WRN: warfarin related nephropathy
INR: international normalized ratio
AKI: acute kidney injury
